# RCN4GSC Meeting Report: Initiating a Testbed for Managing Data at the Interface of Biodiversity and Genomics/Metagenomics, May 2011

**DOI:** 10.4056/sigs.3176515

**Published:** 2012-09-28

**Authors:** Robert J. Robbins, James Beach, Stan Blum, Peter Dawyndt, John Deck, Renzo Kottmann, Norman Morrison, Éamonn Ó Tuama, Inigo San Gil, David Vieglas, John Wieczorek, John Wooley

**Affiliations:** 1 University of California San Diego, La Jolla, California USA; 2 University of Kansas Natural History Museum, Lawrence, KS, USA; 3 Center for Applied Biodiversity Informatics, California Academy of Sciences, San Francisco, California USA; 4 Department of Applied Mathematics and Computer Science, University of Ghent, Ghent, Belgium; 5 Berkeley Natural History Museums, University of California, Berkeley, CA USA; 6 Microbial Genomics Group, Max Planck Institute for Marine Microbiology & Jacobs University Bremen, Bremen, Germany.; 7 School of Computer Science, Kilburn Building, University of Manchester, Oxford Road, Manchester, UK M13 9PL; 8 Global Biodiversity Information Facility, GBIF Secretariat, Copenhagen, Denmark; 9 Department of Biology, LTER Network Office, University of New Mexico, Albuquerque, NM USA; 10 Museum of Vertebrate Zoology University of California Berkeley, CA USA

## Abstract

Following up on efforts from two earlier workshops, a meeting was convened in San Diego to (a) establish working connections between experts in the use of the Darwin Core and the GSC MIxS standards, (b) conduct mutual briefings to promote knowledge exchange and to increase the understanding of the two communities’ approaches, constraints, community goals, subtleties, etc., (c) perform an element-by-element comparison of the two standards, assessing the compatibility and complementarity of the two approaches, (d) propose and consider possible use cases and test beds in which a joint annotation approach might be tried, to useful scientific effect, and (e) propose additional action items necessary to continue the development of this joint effort. Several focused working teams were identified to continue the work after the meeting ended.

## Background

Both the initial Genomic Biodiversity Working Group (GBWG) planning meeting [1] and the follow-up presentation and discussion at the GSC11 meeting [2] called for an effort to bring together expert representatives from the Darwin Core (DwC) community and the GSC MIxS community to compare and analyze the Darwin Core term definitions and the various MIxS checklists, develop a merged checklist approach, and develop test datasets to exercise such a merged approach

## Purposes of the Meeting

**The purposes of the workshop were to:**
- Establish working connections between experts in the use of the Darwin Core and the GSC MIxS standards,
- Conduct mutual briefings to promote knowledge exchange and to increase the understanding of the two communities’ approaches, constraints, community goals, subtleties, etc.,
- Perform an element-by-element comparison of the two standards, assessing the compatibility and complementarity of the two approaches,
- Propose and consider possible use cases and test beds in which a joint annotation approach might be tried to useful scientific effect,
- Propose additional action items necessary to continue the development of this joint effort, and
- Develop an agenda for the time allocated to BDWG at the coming GSC12 meeting in Bremen, Germany.

## Participants

At the initial planning meeting, several attendees made specific recommendations of individuals with DwC expertise who should, if at all possible, be recruited to participate in the joint DwC-GSC analysis. These individuals were contacted and, to a person, they agreed to participate in a joint analysis meeting (the meeting being reported here). Thus, the participants for this meeting were hand picked for their expertise, either with DwC or with GSC standards.

## Activities and Analysis

Recognizing the difficulties for achieving consensus and making appropriate recomendations if there were any disjoint understanding of each other’s methods and approach,^1^ the meeting participants spent most of the first morning presenting, discussing, and analyzing the details of each other’s information systems from scientific, technical, social, and operational perspectives. A major aim for both communities is to avoid reinventing the wheel and instead to understand each other’s methods sufficiently to allow reuse as much as possible.

During the afternoon of the first day, breakout groups proposed and analyzed several candidate use cases, including a proposal to jointly annotate all sequenced bacterial type strains.

One strain — *Shewanella woodyi* — was selected as an example and the group manually produced a description of the strain separately in both GCDML [3] and Simple Darwin Core [4] formats, with a goal of determing whether it would be possible to capture *all* of the terms of interest to *both* communities using only the methods and terms of one or the other community alone. The group determined that this did not work, as not all MIGS mandatory elements could be mapped to DwC (e.g. *submit to insdc*).

**This was not unexpected and served to confirm the need for a *joint* approach to annotation, triggering conversation and speculation on how this might be achieved. For example,**
- Replace GCDML terms with DwC terms,
- Create a DwC Element within GCDML,
- Create a formal Darwin Core Extension based on GCDML,
- Create a SAWSDL [5] based mapping of GCDML elements to DwC, or
- Create alternate schema(s) that pulls from both DwC/GCDML bags of terms.

An examination of joint annotation even led to questions like, “Might metagenomics require alteration of concepts of Taxa and CollectionObject?”

The second day, another breakout group undertook a full, term-by-term comparison of the DwC and GSC checklists. Also, mutual education continued with demonstrations of Ontogrator [6,7] and the use of the DwC Archive [8,9] model for publishing data. Finally, a variety of prototype testbed opportunities were identified and recommended to be pursued (described later).

## Conclusions

The opportunities, both scientific and technical, arising from data management at the biodiversity-(meta)-genomics interface are large and should (must) be pursued. Since it will be impossible to create a single prototype testbed adequate to test all potential solutions, several testbeds (described below) should be pursued simultaneously.

## Recommendations

Interactions should continue between the DwC and GSC communities, spawning collaborative efforts, such as GSC using the DwC-developed Resource Description Framework (RDF) representation of the MIxS checklists. RDF tools can be helpful in the (semi-)automatic production of semantically-aware web sites, thus easing the use of MIxS in the context of the semantic web technologies. Developing a new, independent approach to facilitating the deployment of MIxS checklists in a semantically aware fashion was considered, but this was rejected in favor of a policy of tool re-use, wherever possible. Moreover, the term-by-term break out group came to the conclusion that creating a formal Darwin Core extension would be the most promising first joint approach to data annotation and the most parsimonious way for publishing genome data to GBIF.

**The group also agreed to pursue several prototype testbeds, including**
- develop a Microbial Earth Catalogue,
- explore developing a testbed using Moorea BioCode data (take an entire ecosystem, sequence and take specimens),
- develop MIRADA-LTERS [10] data as a use case of GCDML/EML/DwC harmonization — creating compliant metadata records for MIRADA-LTERs,
- test the development of a use case to publish genome data to GBIF via a Darwin Core Archive (DwC-A) — this is a several step process dependent on the development of orthogonal terms (perhaps benefitting from an RDF representation), then requires discussion with GBIF to frame the goals, scope, and constraints of the experiment, and
- engage NEON/LTER to create a use case based on their needs and data.

Finally, the group recommended that outreach efforts be extended to establish working contact with the fungi-oriented research groups at LTER and to connect with NESCent.

## Timeline for 2011

Efforts by the GBWG to facilitate the development of useful data standards and procedures for the interface of biodiversity with genomics and metagenomics will be an ongoing activity. Here (and in subsequent GBWG reports) we provide a timeline of events. Italics indicate that the suggested activity has already occurred (at the time paper was written); plain text that the activity is proposed.

Mar: *Convene a GBWG planning meeting to initiate an analysis of biodiversity, genomics, and meta-genomics: opportunities and challenges.*

Apr: *Introduce the GBWG initiative at GSC11 meeting, UK; invite the development of use cases.*

May: *Form an RCN Working Group with GSC and Darwin Core specialists*

Jun: Participate in a special session on metagenomics, barcoding, and biodiversity at the iEvoBio meeting to be held 21-22 June 2011 at Norman, OK.

Jul: Engage with DNA barcode standard through Consortium for the Barcode of Life working group. Collect progress reports, assess, and prioritize various testbed projects underway (e.g., Microbial Earth Catalogue. Moorea BioCode. MIRADA-LTERs data sets, publishing genomic data to GBIG using DwC-A, and NEON/LTER.

Sep: Report and discuss progress on initiative at GSC12 meeting, Bremen, Germany.

Oct: Engage GBIF and EOL before and during TDWG meeting, 16-21 October, in New Orleans, Louisiana, US.

Nov: Discuss metadata capture, ecological sampling and analysis, NEON workshop, Boulder, CO.

Dec: Present and discuss initiative at Fourth International Barcode of Life Conference, Adelaide, Australia.
